# Leukocytes from diabetic patients kill retinal endothelial cells: Effects of berberine

**Published:** 2013-10-02

**Authors:** Pei Tian, Hongyan Ge, Haitao Liu, Timothy S. Kern, Lingling Du, Linan Guan, Sheng Su, Ping Liu

**Affiliations:** 1 Eye Hospital, The First Affiliated Hospital, Harbin Medical University, Harbin, China; 2 Departments of Medicine, Pharmacology and Ophthalmology, Case Western Reserve University, and Stokes Veterans Administration Hospital, Cleveland, Ohio

## Abstract

**Purpose:**

Accumulating evidence in animals suggests that leukocytes are involved in the pathogenesis of diabetic retinopathy. The present study was designed to investigate whether leukocytes from diabetic patients could kill retinal endothelial cells and whether that cytotoxicity could be inhibited in vivo by administration of berberine.

**Methods:**

Human retinal endothelial cells (HRECs) were cocultured (24 h) with leukocytes freshly isolated from nondiabetic and diabetic patients, and leukocyte-mediated death of HRECs was analyzed with flow cytometry. HRECs or leukocytes were incubated with antibodies against intercellular adhesion molecule-1(ICAM-1) or integrin beta-2, or with various concentrations of berberine. The protein expression levels of inflammatory factors were investigated using western blots, and activities of antioxidant enzymes and malondialdehyde content were examined as markers of oxidative stress. In addition, leukocytes were isolated from 28 diabetic patients with retinopathy and nondiabetic patients before and after 1 month in vivo therapy with berberine. The effects of the berberine on leukocyte-mediated killing of endothelial cells was again assessed.

**Results:**

Leukocytes from diabetic patients induced more apoptosis of HRECs in a coculture system than did cells from nondiabetic patients, and this killing occurred primarily via direct cell–cell contact. Berberine inhibited the leukocyte-mediated killing of HRECs in vitro, the decrease in antioxidant enzyme activities, the nuclear translocation of nuclear factor kappa B, and the increase in intercellular adhesion molecule-1 and inducible nitric oxide synthase expression and malondialdehyde content in HRECs cultured in high glucose. Berberine also decreased integrin beta-2 expression of leukocytes in vitro and in vivo. Oral consumption of berberine for 1 month likewise inhibited the diabetes-induced increase in leukocyte-mediated killing of HRECs.

**Conclusions:**

Our findings suggest that leukocytes from diabetic patients kill retinal endothelial cells, and that berberine can inhibit this leukocyte-mediated killing of vascular endothelium. Coculture of leukocytes with HRECs might serve as a biomarker to study the role of leukocytes in the development of diabetic retinopathy, and the data are consistent with berberine being a therapy against diabetic retinopathy.

## Introduction

Diabetic retinopathy is one of the most severe microvascular complications of diabetes, and one of the most common causes of blindness in adults. Increasing evidence suggests that increased oxidative stress [1,2] and local inflammation [3] in the retina in diabetes play a significant role in the initiation and development of diabetic retinopathy. Inhibition of oxidative stress by feeding antioxidants or overexpressing antioxidant enzymes inhibits the development of early stages of diabetic retinopathy in animals [4-6]. Recent studies have also indicated that chronic, low-grade inflammation underlies many vascular complications of diabetic retinopathy [3,7], and anti-inflammatory agents have inhibited early stages of the retinopathy in animals [8].

Leukocyte involvement is characteristic of inflammation, and studies of diabetic retinopathy in animals have revealed that leukocytes play a critical role in the development of the vascular complications of diabetic retinopathy, including capillary degeneration and increased vascular permeability [9,10]. Ex vivo, leukocytes from diabetic mice have been shown to kill more retinal endothelial cells than do cells from nondiabetic mice [9], and neutrophils from diabetic animals exhibit greater-than-normal levels of surface integrin expression and integrin-mediated adhesion [11]. Leukocytes use integrin ligand integrin beta-2 (CD18) to tether themselves to intercellular adhesion molecule-1 (ICAM-1) on the surface of diabetic retinal endothelial cells [11]. Whether leukocyte-mediated killing of retinal endothelial cells also occurs in diabetic patients has not been previously reported.

Berberine, a natural extract from Rhizoma coptidis, has been reported for many years as a treatment for intestinal infections. Recently, berberine was found to have also anti-inflammatory, antioxidant, and other effects [12-17]. In diabetes, berberine has been reported to inhibit renal dysfunction via reduction of oxidative stress [18].

In the present study, leukocytes from diabetic patients were found to kill human retinal endothelial cells (HRECs), and berberine administered in vitro or in vivo inhibited the leukocyte-mediated killing of the endothelium. Berberine also had independent beneficial effects on endothelial inflammation and oxidative stress under elevated glucose concentration conditions.

## Methods

### Reagents

For in vitro studies, berberine chloride was purchased from Sigma-Aldrich (St. Louis, MO). The Annexin V: Fluorescein Isothiocyanate Apoptosis Detection Kit was purchased from BD Biosciences (San Jose, CA). The antibodies used in this study included antibodies against human ICAM-1, CD18, nuclear factor of kappa light polypeptide gene enhancer in B-cells inhibitor alpha (IκB-α), p-IκB-α, nuclear factor kappa B (NF-κB) p65, β-actin (Santa Cruz Biotechnology, Paso Robles, CA), and inducible nitric oxide synthase (iNOS; Abcam, Cambridge, MA). Superoxide dismutase (SOD), catalase (CAT), and glutathione peroxidase (GSH-Px) activity kits and a malondialdehyde (MDA) quantification kit were obtained from Jiancheng Bioengineering Institute (Nanjing, China). The NOS activity kit and S-methylisothiourea sulfate (a selective iNOS inhibitor) were purchased from Beyotime Institute of Biotechnology (Shanghai, China).

### Culture of human retinal endothelial cells

Institutional ethics committee approval was obtained, as was informed consent from the first-degree relatives of the donors, for the use of the retinal tissue for research purposes. The donor eyes were obtained and managed in compliance with the Declaration of Helsinki.

Human eyes were obtained from the eye bank within 20 h of the death of the donor. Retinas were detached and digested in an enzyme mixture containing 500 μg/ml collagenase Type I, 200 μg/ml DNase, and 200 μg/ml pronase in phosphate-buffered saline (PBS; 135 mM NaCl, 2.7 mM KCl, 1.5 mM KH_2_PO_4_, and 8 mM K_2_HPO_4_,pH 7.2) for 26–28 min at 37 °C. The suspension was subsequently passed through a 53 μm nylon mesh. The trapped microvessel suspension was resuspended in Dulbecco's Modified Eagle Medium (DMEM)/F12 supplemented with 15% fetal bovine serum, 100 U/ml penicillin and streptomycin, 90 μg/ml heparin, 1% insulin-transferrin-sodium selenite (Gibco BRL, Grand Island, NY), and 5 ng/ml β-endothelial cell growth factor (Peprotech, Rocky Hill, NJ). The microvessel fragments were placed in culture flasks that had been coated with 0.2% gelatin and were incubated in 5% CO_2_ at 37 °C. HRECs were passaged after 8–10 days of primary culture when large confluent areas were observed. The second passage of HRECs was plated on chamber slides to be characterized with platelet/endothelial cell adhesion molecule-1 antibody (PECAM-1, Santa Cruz Biotechnology) via immunocytochemistry. A human retinal endothelial cell preparation that was >90% positive for PECAM-1 immunostaining was considered acceptable for the study. Cells from passages 2 and 5 that displayed 80% conﬂuency were used for the experiments.

### Leukocyte isolation

Peripheral blood was obtained from diabetic and control patients from cubital vein using EDTA tubes and was preserved at 4 °C prior to use. Leukocytes were isolated using discontinuous Percoll density gradient centrifugation [19] according to the manufacturer’s instruction (GE Healthcare, Beijing, China) within 2 h after the peripheral blood was obtained. Isolated cells were washed and resuspended in PBS. This procedure yielded 95% pure polymorphonuclear cells (based on acetic acid-crystal violet staining), and the population was 98% viable (determined with trypan blue exclusion). Cells were used immediately after separation for coculture.

### Human retinal endothelial cells and leukocyte coculture

HRECs were cultured in low-glucose medium (L, 5.5 mM) or high-glucose medium (H, 25 mM) for 48 h before being cocultured with leukocytes. Unstimulated leukocytes from ten nondiabetic patients (N, five men and five women, average age of 59±10 years) and ten type 2 diabetic patients with retinopathy (D, five men and five women, average age of 62±9 years old, diabetes duration of 14±2 years, fasting blood glucose (FBG) 9.9±0.4 mmol/l; all diabetic patients were insulin treated) were resuspended at 1×10^5^ cells/ml, and cocultured with HRECs at a ratio of 1:5 in low- or high-glucose medium with direct contact for an additional 24 h.

To differentiate the effect of the cell adhesion of leukocytes to HRECs from those due to the release of soluble factors from the leukocytes, transwell assays were performed in parallel. HRECs were grown on transwells with microporous membrane inserts (0.4 μm pore size, Falcon, Paramus, NJ) that physically separated the leukocytes from the endothelial cells. Other conditions were identical to the experiments described above.

Some HRECs were cultured in high glucose for 48 h before being treated with anti-ICAM-1 antibody (1:100 dilution) for 12 h in high-glucose DMEM/F12 with 15% FBS. Others were preincubated with various concentrations (0 μM, 5 μM, 25 μM, 50 μM, and 100 μM) of berberine for 24 h, and subsequently cultured in high-glucose medium for another 48 h. HRECs cultured in low-glucose medium served as a control. Some leukocytes from diabetic patients were preincubated with anti-CD18 antibody (1:100 dilution) for 12 h in high-glucose Rosewell Park Memorial Institute (RPMI) 1640 with 5% FBS, and others were preincubated with various concentrations (0 μM, 5 μM, 25 μM, 50 μM, and 100 μM) of berberine for 24 h. The activation of the NF-κB signaling pathway was investigated after a 12 h treatment with berberine followed by incubation in high glucose for 24 h.

### Apoptosis assay

After coculture, apoptosis of HRECs was measured with flow cytometry. The cells were washed with PBS, resuspended in binding buffer, and incubated with P-phycoerythrin (PE)-coupled CD144 antibody (to label the HRECs) for 15 min, followed by incubation with Annexin V and propidium Iodide (PI; to identify dead or damaged cells) for 15 min according to the manufacturer’s instruction (BD Biosciences, San Jose, CA). The cells were analyzed on a flow cytometer to measure apoptosis.

### Neutrophil–endothelial adhesion assay

Leukocytes from diabetic patients were preincubated with various concentrations (0 μM, 5 μM, 25 μM, 50 μM) of berberine for 24 h. To label the leukocytes, 2×10^7^ cells/ml was incubated with 40 μg/ml calcein-AM (Sigma-Aldrich) for 30 min at 37 °C [20]. Calcein-AM is nontoxic and has no effect on cell adhesion [21]. The labeled cells were washed and resuspended in RPMI 1640. HRECs were preincubated with various concentrations (0 μM, 5 μM, 25 μM, 50 μM) of berberine for 24 h, and subsequently cultured in high-glucose medium for another 48 h. The suspension of labeled neutrophils was added to the monolayer of endothelial cells (2×10^6^ leukocytes/well) at a ratio of 1:5 for 90 min at 37 °C. After incubation, non-adherent cells were removed by gentle washing with RPMI. The emitted fluorescence of the adherent leukocytes was measured in a fluorescence plate reader (excitation wavelength, 490 nm; emission wavelength, 530 nm).

### Western blot analysis

Following coculture, HRECs were homogenized in protein lysate buffer, and debris was removed by centrifugation. The protein content was determined, and the samples were resolved on 12% polyacrylamide–sodium dodecyl sulfate (SDS) gels before being electrophoretically transferred to polyvinylidene diﬂuoride membranes. The membranes were blocked with 5% BSA; incubated with primary antibodies against ICAM-1, IκB-α, p-IκB-α, NF-κB p65, β-actin, or iNOS; and subsequently probed with an alkaline phosphatase-conjugated secondary antibody (Santa Cruz Biotechnology). The membranes were then developed with 5-bromo-4-chloro-3-indolyl phosphate/nitro blue tetrazolium.

### Assay of antioxidant enzyme activities and malondialdehyde content in human retinal endothelial cells

Antioxidant enzyme activities and MDA content were measured in each group. Superoxide dismutase activity was assayed by measuring the degree of inhibition of 4-nitro-blue tetrazolium chloride (NBT) using the xanthine-xanthine oxidase system to generate superoxide anions [22]. Catalase activity was assayed with a direct measurement of H_2_O_2_ decomposition [23]. GSH-px activity was measured as the rate of glutathione oxidation catalyzed by the GSH-px present in the supernatant, with H_2_O_2_ as the substrate [24]. The MDA content was estimated using the thiobarbituric acid reagent method [25]. All experiments were performed according to the manufacturer’s instruction.

### Assay of inducible nitric oxide synthase enzymatic activity

HRECs were preincubated in low-glucose medium with various concentrations (0 μM, 5 μM, 25 μM, 50 μM) of berberine for 24 h, and subsequently cultured in high-glucose medium with various concentrations of berberine for another 12 h in 96-well plate. HRECs cultured in low-glucose medium served as a control. The culture supernatant was removed, and 100 μl of NOS assay buffer (1×) with or without 0.1 mM S-methylisothiourea sulfate were added to each well. Then 100 μl of NOS assay reaction solution (50% NOS assay buffer, 39.8% MilliQ water, 5% L-Arginine solution, 5% 0.1 mM nicotinamide adenine dinucleotide 2'-phosphate reduced tetrasodium salt (NADPH), 0.2% 3-Amino,4-aminomethyl-2',7'-difluorescein, diacetate [DAF-FMDA]) was added to each well and incubated for 2 h at 37 °C. Fluorescence was measured with a fluorescence plate reader at excitation of 495 nm and emission of 515 nm [26].

### Estimation of integrin beta-2 expression

The surface expression of CD18 on peripheral blood leukocytes was determined with flow cytometry. Briefly, 1×10^5^ cells were cultured in 0.5 mg/ml isotype control or anti-CD18 antibodies (Santa Cruz Biotechnology), incubated for 30 min on ice, and then washed with PBS. The cells were subsequently incubated in 0.5 mg/ml fluorescein isothiocyanate–labeled second antibody (Santa Cruz Biotechnology) for 30 min on ice. After washing and resuspension in 500 μl of PBS, the fluorescence of 1×10^4^ cells was measured with flow cytometry. Leukocytes were manually gated based on their characteristic forward and side light-scattering properties. Surface expression was presented as the mean channel fluorescence on a logarithmic scale. The percentage of the CD18-positive leukocytes was evaluated.

### In vivo effect of berberine

The study was approved by the Ethics Committee of the First Affiliated Hospital of Harbin Medical University, China, and informed consent was obtained after the purpose of the study was fully explained to each subject. This clinical trial was registered on the Chinese Clinical Registry (registration number: ChiCTR-TRC-12002898).

The study enrolled 28 insulin-treated type 2 diabetic patients with diabetic retinopathy (13 men and 15 women, average age of 63±9 years old, diabetes duration of 14±2 years, FBG 10.0±0.4 mmol/l). The exclusion criteria were any endocrine disease other than diabetes, severe hypertension (blood pressure >180/110 mmHg), recent use of anti-inflammatory drugs, hepatitis, or rheumatologic or neoplastic diseases, and for the diabetics, diabetes duration of less than 10 years. Subjects with diabetic retinopathy were assigned to take berberine hydrochloride (Shengyang First Pharmaceutics, Shenyang, China) orally at a dose of 0.5 g twice a day for 1 month. Venous blood samples were taken from each subject, before and after berberine therapy in the treatment group. Peripheral blood was obtained from cubital vein using EDTA tubes from each subject, before and after berberine therapy in the treatment group, and was preserved at 4 °C prior to use.

Leukocytes were isolated from diabetic patients before and after oral berberine therapy, and were cocultured with HRECs in direct contact for an additional 24 h in high glucose as described above. Leukocyte-mediated killing of HRECs was studied as described above.

### Statistical analysis

Statistical analyses were performed with SPSS for Windows version 17.0 (SPSS, Chicago, IL). All the data are expressed as the mean values ± standard deviation (SD). A two-way analysis of variance (ANOVA) was conducted to examine the effect of the high-glucose condition and leukocytes from diabetic patients on HREC apoptosis. Comparisons among multiple groups in other experiments were made with ANOVA followed by a Dunnett test. To compare the values before and after berberine treatment, a paired Student *t* test was used. p<0.05 was considered statistically significant.

## Results

### Leukocytes from diabetic patients induce human retinal endothelial cell apoptosis by direct contact

**Figure 1:**
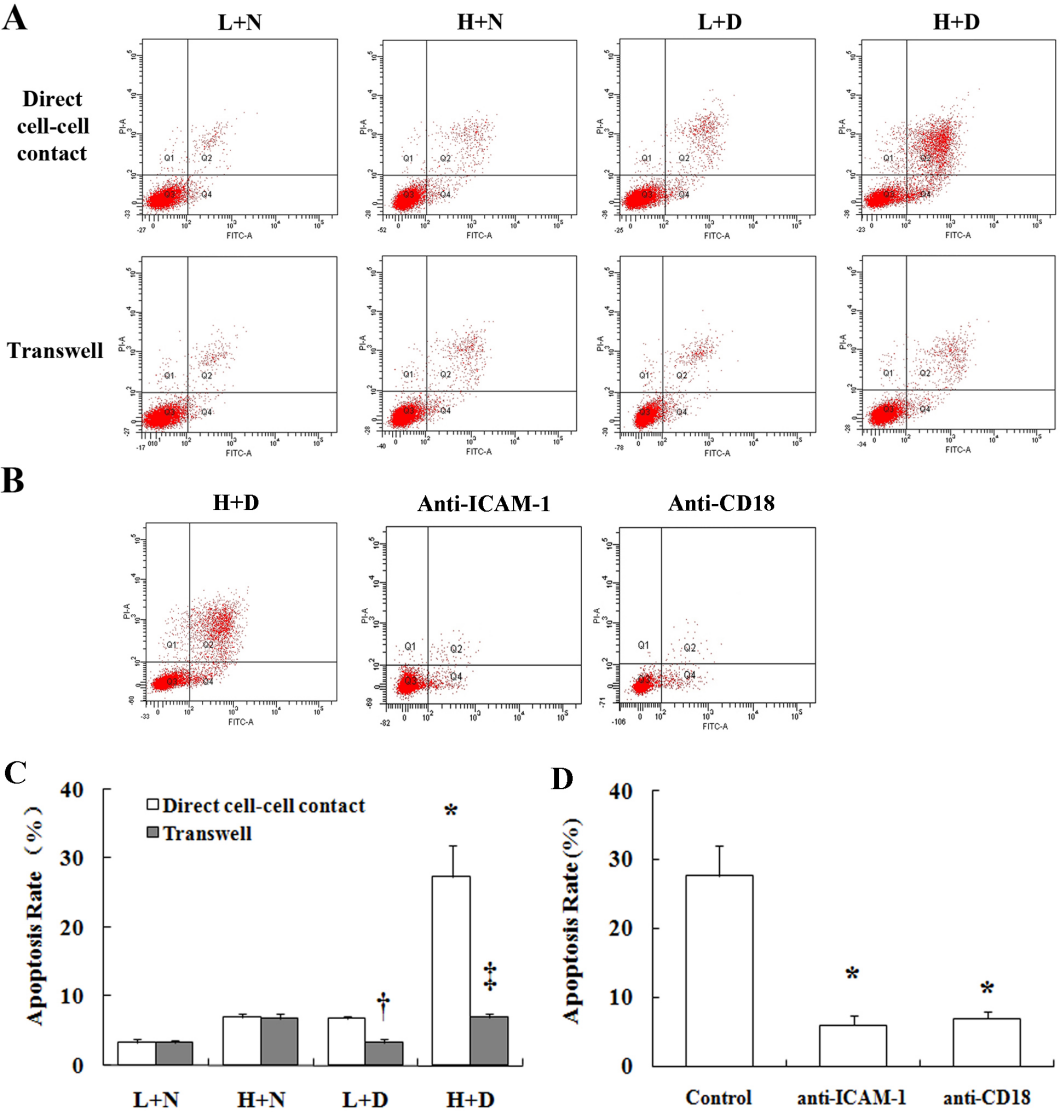
Leukocytes from diabetic patients induce death of human retinal endothelial cells (HRECs). **A**: Comparison of endothelial death due to direct contact between leukocytes and human retinal endothelial cells (HRECs) and release of soluble factors from leukocytes are shown. Representative dot plots from cytometrically analyzed cells cultured in various coculture systems are shown. L, low-glucose medium; H, high-glucose medium; N, neutrophils from nondiabetic patients; D, neutrophils from diabetic patients. **B**: Inhibiting contact of leukocytes with HRECs using antibodies against intercellular adhesion molecule-1 (ICAM-1) or integrin beta-2 (CD18) inhibits endothelial death in the H+D coculture system. **C**: Graphical summary of HREC apoptosis from data in 1A is shown (n=10). * indicates that the effect of high glucose and neutrophils from diabetic patients on HREC apoptosis was significant (p<0.001). ^†^ indicates a significant decrease in the apoptosis rate in the transwell system versus direct cell–cell contact in the L+D group (p<0.05), and ^‡^ indicates significantly less apoptosis in the transwell system versus direct cell–cell contact in the H+D group (p<0.05). **D**: Graphical summary of HREC apoptosis from data in 1B (n=3). * indicates a significant difference (p<0.001) compared to the non-treated group. Error bars represent SD.

To determine whether leukocytes from diabetic patients induce apoptosis in retinal vascular cells, leukocytes derived from nondiabetic and diabetic patients were cocultured with HRECs followed by flow cytometry. As shown in Figure 1, addition of leukocytes from nondiabetic patients had only a modest effect on the death of endothelial cells. In contrast, leukocytes from diabetic patients caused much more death of HRECs than did leukocytes from nondiabetic controls or HRECs incubated solely in high glucose (in the absence of leukocytes). The combination of high glucose in media plus leukocytes from diabetic patients gave the greatest amount of HREC death. Statistical analysis of these results demonstrated that the effects of high glucose and leukocytes from diabetic patients on the HREC apoptosis rate were significant (p<0.001), and the interaction between these factors had statistical significance (p<0.001).

We next used a transwell system to determine if the leukocytes killed endothelial cells via the release of soluble factors (Figure 1). HRECs were cultured in the upper chamber of the inserts (0.4 μm pore size) and the leukocytes in the lower wells. Direct cell–cell contact is not allowed, but soluble factors can be freely exchanged. These assays showed that factors released from leukocytes isolated from diabetic patients had only a little ability to induce HREC apoptosis (the amount of change was not statistically significant). The apoptosis rates of the L+N, H+N, L+D, and H+D groups in the transwell assay were 3.3±0.3%, 6.9±0.7%, 3.3±0.4%, and 7.1±0.4%, respectively. Apparently, cell adhesion or at least close contact is required for leukocytes from diabetic patients to induce HREC apoptosis.

Anti-ICAM-1 and anti-CD18 antibodies were used to block direct contact between HRECs and leukocytes in the direct cell–cell contact coculture system. As shown in Figure 1B,D, the HREC apoptosis rate with leukocytes from diabetic patients decreased significantly from the control value of 27.6±4.5% to 6.1±1.3% and 6.9±1.1% (both p<0.05) when the cells were preincubated with anti-ICAM-1 or anti-CD18 antibody, respectively. The findings support the concept that leukocyte–endothelial contact is important for leukocyte-mediated killing of HRECs in diabetes.

### Berberine inhibits leukocyte-mediated apoptosis of human retinal endothelial cells in vitro

**Figure 2:**
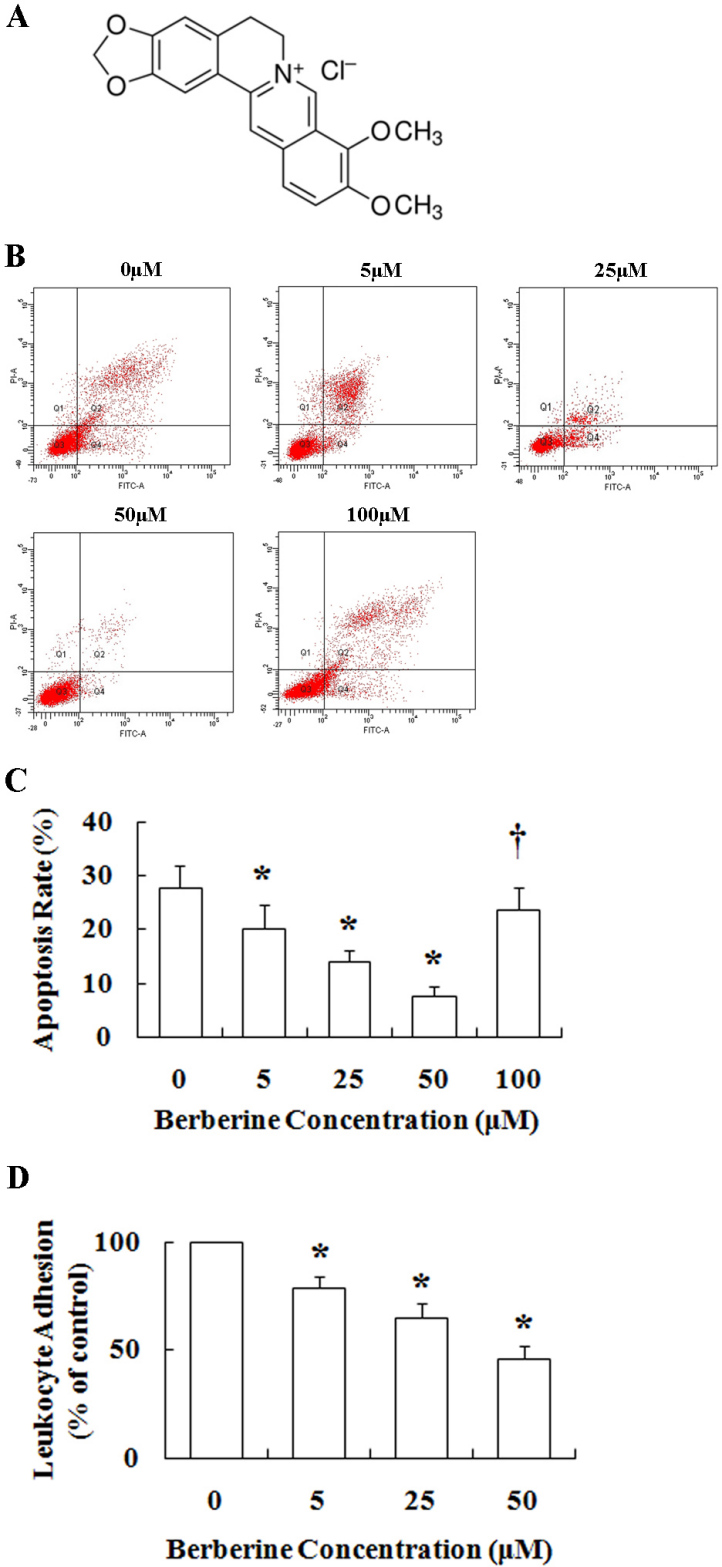
Berberine inhibited leukocyte-mediated human retinal endothelial cells (HRECs) apoptosis in vitro. **A**: Molecular structure of berberine hydrochloride is shown. **B**: Dot plots represent human retinal endothelial cell (HREC) apoptosis caused by leukocytes. HRECs and leukocytes were pretreated with various concentrations of berberine (0–100 μM), and subsequently cultured in the H+D coculture system. **C**: Graphical summary of HREC apoptosis from data in 2B is shown (n=3). * p<0.001 compared to the non-treated group; ^†^ indicates p<0.001 compared to the 50 μM berberine group. **D**: Berberine inhibited leukocyte adhesion to HRECs in high-glucose medium. * p<0.05 compared to the non-treated group.

HRECs and leukocytes were preincubated with various concentrations of berberine (0 μM, 5 μM, 25μM, 50 μM, 100 μM) before being cocultured. As shown in Figure 2, the apoptosis of HRECs incubated with leukocytes decreased significantly from the control value (in high glucose; 27.8±3.9%) as the concentration of berberine increased (20.2±4.3%, 14.1±2.0%, and 7.6±1.6% ; all p<0.05) when the cells were preincubated with 5 μM, 25 μM, or 50 μM berberine, respectively. However, the HREC apoptosis rate increased to 23.6±4.2% when the cells were preincubated with 100 μM berberine. Thus, berberine inhibited leukocyte-mediated apoptosis in a dose-dependent manner from 5 μM to 50 μM but began to show toxicity at 100 μM.

### Berberine inhibits leukocyte adhesion to human retinal endothelial cells in vitro

As shown in Figure 2D, the leukocyte adhesion rate decreased significantly after the leukocytes were incubated with berberine. The adhesion rates were 79.0±4.9%, 64.7±6.6%, and 45.7±6.2%, when the HRECs and leukocytes were pretreated for 24 h with 5 μM, 25 μM, or 50 μM of berberine, respectively, compared with the control group without berberine incubation.

### Berberine decreases intercellular adhesion molecule-1 and integrin beta-2 expression

**Figure 3:**
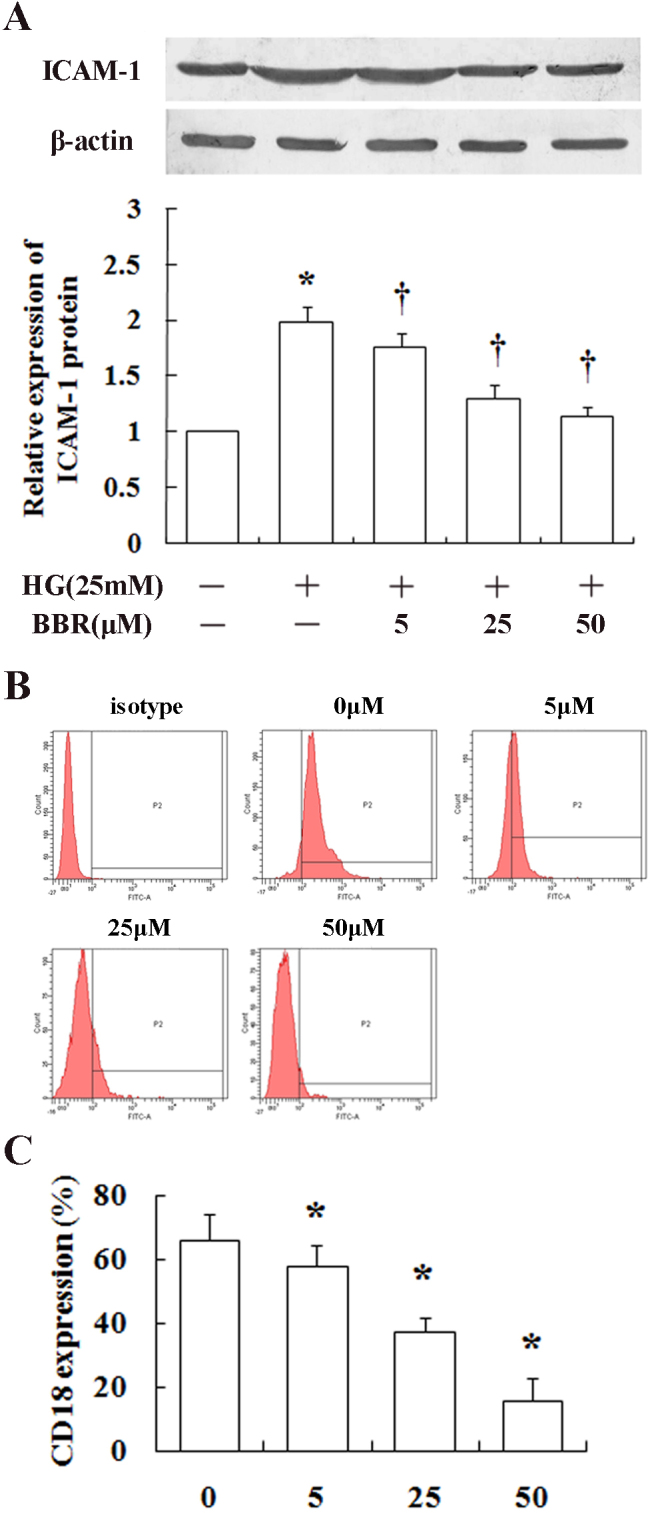
Effects of berberine on protein expression intercellular adhesion molecule-1 (ICAM-1) and integrin beta-2 in high glucose. **A**: Quantification of ICAM-1 expression on human retinal endothelial cells (HRECs) from various treatment groups by western blot is shown. The data in (**B**) are expressed as cell counts (y-axis) plotted as a function of fluorescence intensity (x-axis) and are representative of independent experiments in each group. The histogram (**C**) represents the mean intensities of anti-integrin beta-2 (CD18) from flow cytometric analysis, normalized to the immunoglobulin (IgG) isotype control. * p<0.05 between low- and high-glucose-treated cells; ^†^ p<0.05 between berberine-treated and non-treated cells cultured in high glucose. The results are representative of three independent experiments.

To investigate the mechanism of berberine to inhibit leukocyte-mediated apoptosis of HRECs, ICAM-1 expression in HRECs and CD18 expression in leukocytes were evaluated. As shown in Figure 3A, treatment with high glucose (25 mM) significantly increased the expression of ICAM-1 in HRECs (p<0.05 compared to the normal glucose group), and berberine inhibited high glucose-induced expression of ICAM-1 in a dose-dependent manner with a maximal inhibitory effect achieved at 50 μM. Berberine also decreased CD18 expression in leukocytes from diabetic patients in vitro in a dose-dependent manner (Figure 3B,C).

### Berberine inhibits nuclear factor kappa B signaling activation in human retinal endothelial cells incubated in high glucose

**Figure 4:**
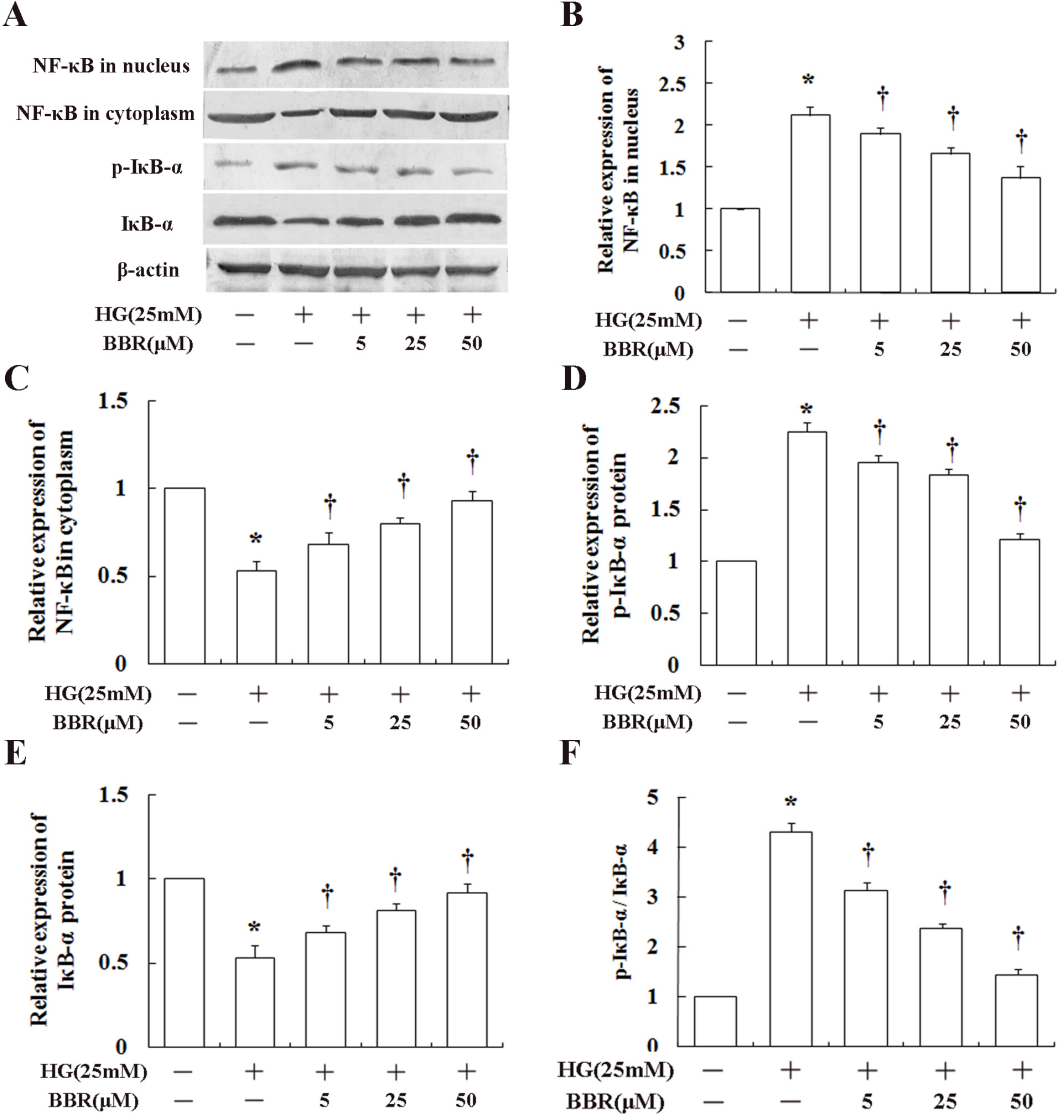
Effects of berberine (BBR) on nuclear factor kappa B (NF-κB) signaling pathway activation were shown. Human retinal endothelial cells (HRECs) were pretreated with 5, 25, or 50 μM berberine for 12 h before being exposed to high glucose for 24 h. **A**: Representative results of western blot were shown. Berberine inhibited high-glucose-induced (**B**) accumulation of nuclear factor kappa B (NF-κB) p65 expression in the nucleus and (**C**) increased p65 expression in the cytoplasm. **D**–**E**: Berberine inhibited high-glucose-induced phosphorylation of a key enzyme involved in activation of NF-κB (nuclear factor of kappa light polypeptide gene enhancer in B-cells inhibitor, alpha; IκB-α) and the degradation of IκB-α, resulting in (**F**) a significant effect on the p-IκB-α/IκB-α ratio. The results are representative of three independent experiments, and are summarized graphically in **B**–**F**. The density of each band was measured and compared to that of the internal control, β–actin. * indicates a significant difference (p<0.05) in band density between low- and high-glucose-treated cells; ^†^ indicates a significant difference (p<0.05) in band density between berberine-treated and non-treated cells cultured in high glucose.

To study the mechanism underlying the anti-inflammatory effects of berberine on HRECs under high-glucose conditions in vitro, we explored the effect of berberine on NF-κB translocation to the nucleus using western blot analysis. As shown in Figure 4, high glucose significantly increased NF-κB p65 in nuclear extracts of HREC expression by approximately twofold compared to normal glucose. In contrast, pretreatment with 5, 25, or 50 μM berberine for 12 h exerted a dose-dependent inhibition of p65 levels in the nucleus (and decreasing levels in cytoplasm) compared with the high-glucose treatment group (p<0.05; Figure 4A–C). Furthermore, high glucose significantly decreased IκB-α expression levels in the cytoplasm, and pretreatment with berberine for 12 h markedly inhibited the glucose-induced reduction in expression of IκB-α (Figure 4D,E). Consistent with this, berberine strongly inhibited phosphorylation and degradation of IκB-α.

### Berberine reduces oxidative stress in human retinal endothelial cells incubated in 25 mM glucose

**Figure 5:**
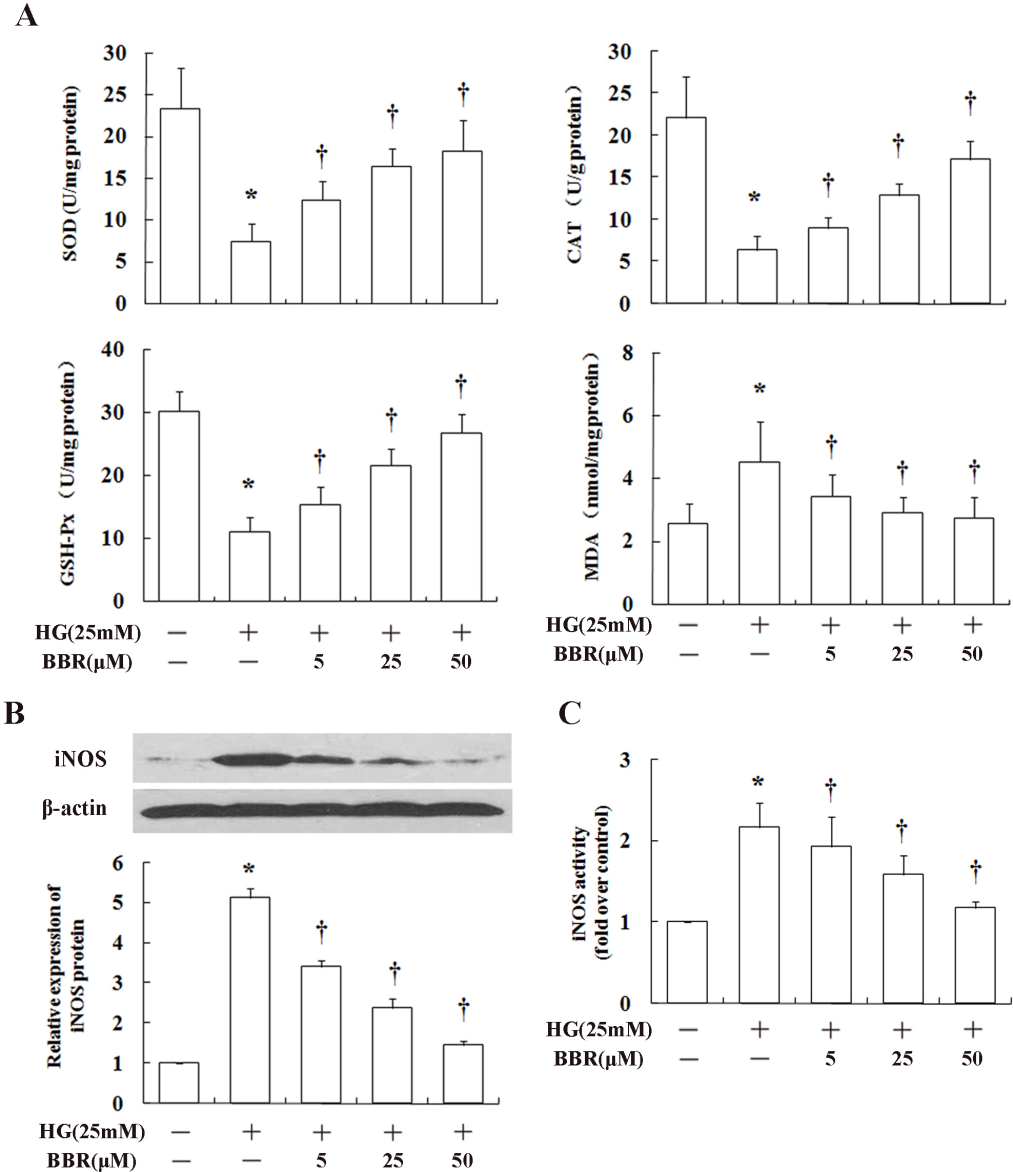
Effects of berberine on oxidative stress in human retinal endothelial cells (HRECs). Berberine (**A**) restored antioxidant enzyme activities including superoxide dismutase (SOD), catalase (CAT), and glutathione peroxidase (GSH-Px) and reduced malondialdehyde (MDA) content, (**B**) inhibited expression of inducible nitric oxide synthase (iNOS), and (**C**) decreased iNOS activity in high glucose. The density of each band was measured and compared to that of the internal control, β–actin. * p<0.05 between low-glucose- and high-glucose-treated cells; ^†^ p<0.05 between berberine-treated and non-treated cells cultured in high glucose.

As shown in Figure 5, SOD, CAT, and GSH-px activity decreased dramatically when HRECs were cultured in high glucose, whereas MDA concentration increased significantly. After preincubation in berberine, the glucose-induced loss of antioxidant enzyme activities in the HRECs (SOD, CAT, and GSH-px) and increase in MDA were inhibited. iNOS, which influences oxidative stress and inflammation, is regulated by NF-ĸB, and iNOS expression likewise was inhibited in a dose-dependent manner by berberine. Furthermore, the effect of berberine on the activity of iNOS was examined. HRECs were treated with high glucose with or without the indicated concentrations of berberine. As shown in Figure 5C, high glucose caused a twofold increase in iNOS enzymatic activity. Berberine strongly inhibited the iNOS enzymatic activation in HRECs in a dose-dependent manner.

### In vivo administration of berberine inhibits the diabetes-induced killing of human retinal endothelial cells by leukocytes and decreases integrin beta-2 expression on leukocytes

**Figure 6:**
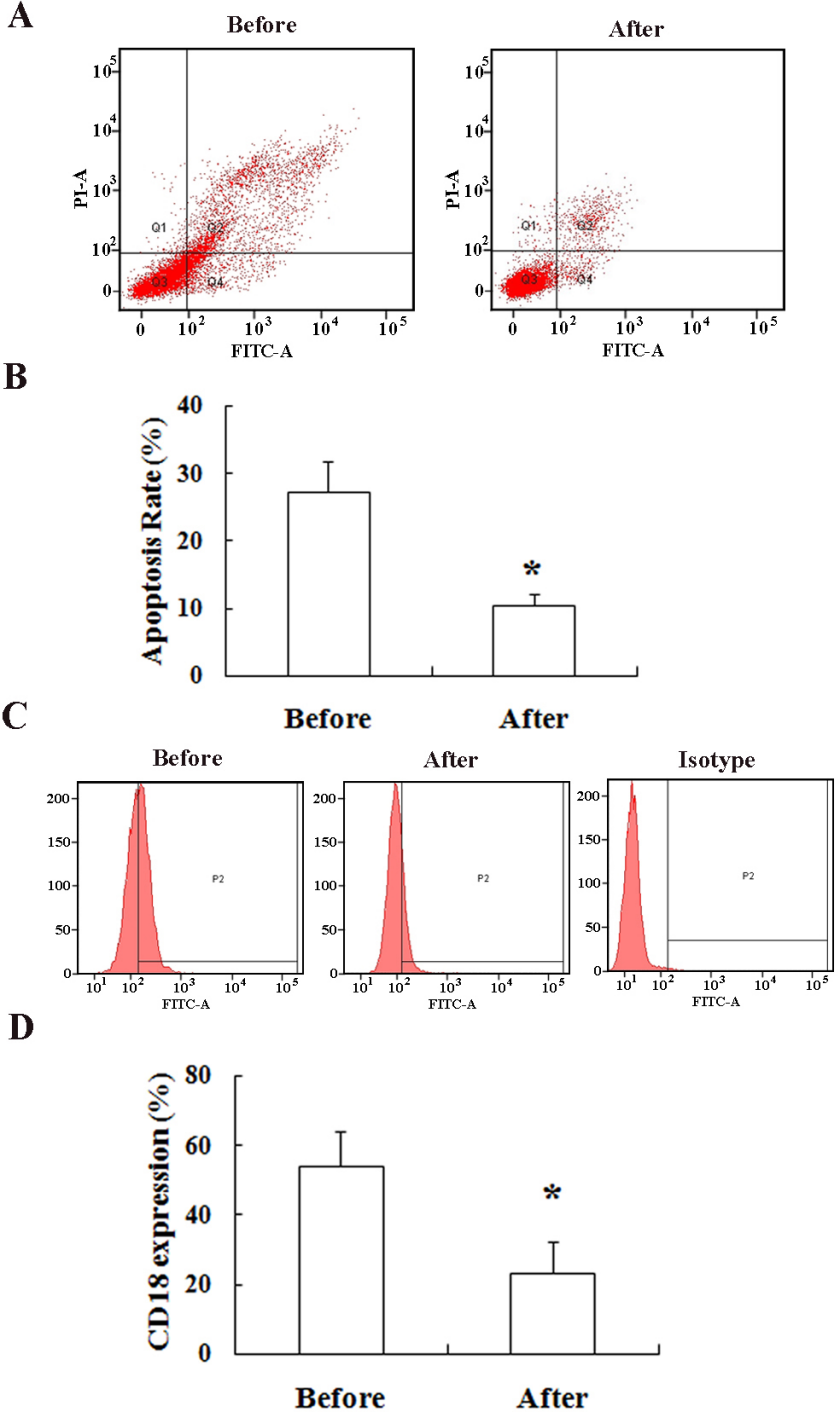
Berberine taken orally by diabetic patients inhibited leukocyte-mediated apoptosis of human retinal endothelial cells (HRECs; **A** and **B**) and decreased integrin beta-2 (CD18) expression on neutrophils (**C** and **D**). **A** and **B** show representative dot plots of leukocyte-mediated death of HRECs before and after berberine treatment. The data in **C** are expressed as cell counts (y-axis) plotted as a function of fluorescence intensity (x-axis), and show CD18 expression and leukocyte-mediated killing of HRECs before and after the 1 month treatment with berberine. Plots shown are representative of independent experiments in each group. Results in the histogram of CD18 expression (**D**) are normalized to the immunoglobulin (IgG) isotype control. * Leukocyte-mediated killing of HRECs after berberine is p<0.05 compared to the same measurement before berberine treatment. N=28.

Leukocytes were collected from another group of nondiabetic and diabetic patients, and the cells were cocultured with HRECs as described above. After 1 month of daily treatment with berberine, leukocytes were again collected from the same patients. The diabetes-induced killing of HRECs by leukocytes was remeasured, and was significantly inhibited. Comparing the leukocyte-mediated killing of HRECs before and after the berberine treatment, the HRECs killed by leukocytes from diabetic patients decreased from 27.3±4.5% before the berberine therapy to 10.4±1.7% after the berberine therapy (p<0.05). The average CD18 expression decreased significantly from 53.9±9.8% before the berberine treatment to 23.2±9.0% after the 1 month oral berberine treatment (p<0.05; Figure 6).

As reported previously [27], berberine therapy also significantly reduced glycemia. During the 1 month of therapy, glycosylated hemoglobin (HbA_1_C) decreased from 8.2±0.3% to 7.0±0.6% (p<0.05).

## Discussion

Mounting evidence suggests that inflammatory-like changes and oxidative stress are integrally involved in the development of diabetic retinopathy [1-3]. Regarding inflammation, corticosteroids (one of the most powerful anti-inflammatory agents known) had dramatic effects on inhibiting retinal edema in diabetes [28-30]. In addition, various proinflammatory proteins are enriched in the retina and vitreous from diabetic patients [31-34]. Animal studies have shown that inhibiting local proinflammatory mediators inhibits the development of early stages of diabetic retinopathy, including the degeneration of retinal capillaries and increased permeability [1,3,4,8,35]. Regarding oxidative stress, long-term administration of antioxidants or overexpressing antioxidant enzymes inhibits the development of the early stages of the retinal microvascular disease in animals [4-6]. Leukocytes play important role in both abnormalities in the retina in diabetes, because deleting even a single gene from the leukocytes of diabetic mice has been shown to inhibit the diabetes-induced increase in inflammatory proteins and superoxide generation by the retina [9,10].

Our previous studies show that leukocytes from diabetic mice [9,10], and now leukocytes from diabetic patients, cause direct injury and death of retinal endothelial cells. The ex vivo coculture assays using leukocytes from diabetic patients showed that killing of HRECs by leukocytes from diabetic patients largely depends on direct contact, which indicates that cell surface molecules are involved. Blocking the adhesion molecule, ICAM-1 or CD18, with specific antibodies in the present studies inhibited the leukocyte-mediated death of HRECs, and deletion of these proteins from diabetic mice previously inhibited the diabetes-induced degeneration of retinal capillaries in vivo [7]. Leukocyte adhesion to endothelial cells leads to Fas-FasL pathway activation, which can result in Fas-mediated apoptosis and blood–retinal barrier breakdown [36]. Thus, therapies that block leukocyte-endothelial cell–cell contact should help inhibit endothelial cell death in diabetic retinopathy (and perhaps at other sites of the microvascular disease).

Berberine is known to have anti-inflammatory and antioxidant effects [13,16-18]. We postulated that berberine therefore might be effective for treating diabetic retinopathy. In the present study, berberine inhibited diabetes-induced killing of HRECs by leukocytes although the leukocytes from diabetic patients had been chronically exposed to hyperglycemia in vivo. Even during the short interval of incubation in vitro, berberine significantly inhibited leukocyte adhesion to HRECs as well as the leukocyte-mediated killing of endothelial cells. One likely mechanism by which berberine inhibits leukocyte-mediated killing of HRECs is via downregulation of ICAM-1 (on endothelial cells) and CD18 (on leukocytes), both of which were inhibited by berberine in the present study.

To study the mechanism underlying the anti-inflammatory and antioxidant effects of berberine on HRECs, one important signaling pathway that regulates inflammation was investigated. NF-κB initiates transcription of various inflammatory genes (including ICAM-1 and iNOS), and activation of NF-κB can occur by phosphorylation-mediated degradation of IκB-α, an endogenous inhibitory protein of NF-κB. Our results suggest that berberine performed its anti-inflammatory effects, at least in part, by regulating NF-κB activation in the endothelial cells.

Liu et al. reported that berberine could ameliorate renal dysfunction in diabetic nephropathy by reducing oxidative stress [18]. Consistent with these findings, our study showed that levels of important antioxidant enzymes (SOD, CAT, and GSH-Px) in the retina became subnormal in HRECs exposed to elevated glucose, and that berberine treatment inhibited the loss of those activities. Berberine also inhibited the accumulation of MDA in HRECs cultured in high glucose. Thus, it seems likely that berberine can exert its beneficial effect via multiple pathways.

The potential for berberine to have therapeutic benefit in diabetic patients seems clear, so we tested it in vivo. Our in vitro studies suggested toxicity at the highest concentration of berberine, but prior studies demonstrated the safety of oral berberine in patients [17,26,37]. In 14 clinical studies involving 1,068 patients with type 2 diabetes, no adverse effects were noted at doses of berberine between 0.5 g and 1.5 g per day (total daily intake was divided into two or three doses) [38]. In our study, we administered a dose of 0.5 g berberine twice a day, and no adverse effects were reported. Measurement of leukocyte-mediated killing of HRECs before and after 1 month treatment with berberine demonstrated that the treatment dramatically inhibited killing of endothelial cells by leukocytes from diabetic patients. Since this cytotoxic effect of leukocytes on endothelial cells has been closely linked to degeneration of retinal capillaries and other lesions of early diabetic retinopathy [9,10], the findings suggest that berberine or other agents with similar activity might inhibit the development of retinopathy.

In contrast to the in vitro studies, the interpretation of how berberine works in vivo is confounded by an effect of berberine to reduce glycemia. Thus, berberine in vivo might inhibit endothelial cell death by leukocytes directly via inhibition of inflammation/oxidative stress, as well as by improvement in glycemia.

In conclusion, the present study demonstrates that leukocytes from diabetic patients induce apoptosis in retinal endothelial cells by direct cell–cell contact, and provide evidence in humans that leukocytes might play an important role in the development of diabetic retinopathy. This leukocyte-mediated killing of endothelial cells can be modeled in vitro, and a coculture system should be explored as a possible biomarker for susceptibility to diabetic retinopathy. Our studies further demonstrate that therapeutic use of berberine significantly inhibited this leukocyte-mediated killing of retinal endothelial cells and inhibited the induction of markers of inflammation and oxidative stress in elevated glucose. The mechanism underlying the inhibitory effects of berberine on inflammation and oxidative stress is mediated, at least partly, by blocking activation of the NF-κB signaling pathway. Berberine and related molecules should be tested for their ability to inhibit diabetic retinopathy.
